# 1-[4-(4-Hy­droxy­phen­yl)piperazin-1-yl]ethanone

**DOI:** 10.1107/S1600536813028031

**Published:** 2013-10-19

**Authors:** Channappa N. Kavitha, Jerry P. Jasinski, Brian J. Anderson, H. S. Yathirajan, Manpreet Kaur

**Affiliations:** a Department of Studies in Chemistry, University of Mysore, Manasagangotri, Mysore 570 006, India; b Department of Chemistry, Keene State College, 229 Main Street, Keene, NH 03435-2001, USA

## Abstract

In the title compound, C_12_H_16_N_2_O_2_, the piperazine ring has a chair conformation. The dihedral angle between the mean planes of the benzene ring and the acetyl group is 48.7 (1)°. In the crystal, mol­ecules are linked *via* O—H⋯O hydrogen bonds, forming chains propagating along [010].

## Related literature

For the biological activity of piperazine derivatives, see: Bogatcheva *et al.* (2006 ▶); Brockunier *et al.* (2004 ▶); Elliott (2011 ▶); Kharb *et al.* (2012 ▶). For the crystal structures of related compounds, see: Dayananda *et al.* (2012 ▶); Kavitha *et al.* (2013*a* ▶,*b* ▶); Peeters *et al.* (1979 ▶, 2004 ▶). For puckering parameters, see: Cremer & Pople (1975 ▶). For standard bond lengths, see: Allen *et al.* (1987 ▶).
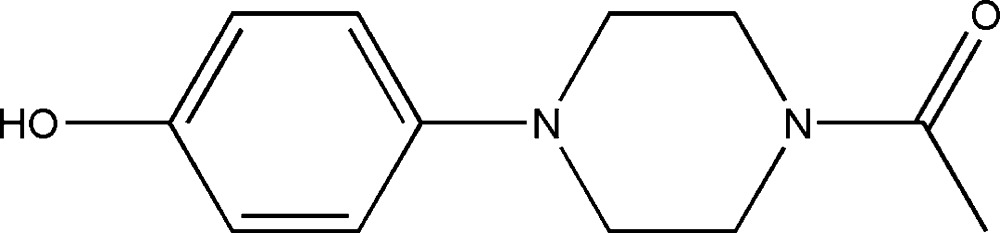


## Experimental

### 

#### Crystal data

C_12_H_16_N_2_O_2_
*M* *_r_* = 220.27
Monoclinic, 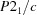

*a* = 6.13183 (19) Å
*b* = 12.0106 (4) Å
*c* = 14.8704 (5) Å
β = 94.025 (3)°
*V* = 1092.46 (6) Å^3^
*Z* = 4
Cu *K*α radiation
μ = 0.75 mm^−1^
*T* = 173 K
0.48 × 0.46 × 0.32 mm

#### Data collection

Agilent Xcalibur (Eos, Gemini) diffractometer
Absorption correction: multi-scan (*CrysAlis PRO* and *CrysAlis RED*; Agilent, 2012 ▶) *T*_min_ = 0.833, *T*_max_ = 1.000
6224 measured reflections
2134 independent reflections
1944 reflections with *I* > 2σ(*I*)
*R*_int_ = 0.025

#### Refinement

*R*[*F* ^2^ > 2σ(*F* ^2^)] = 0.041
*wR*(*F* ^2^) = 0.113
*S* = 1.07
2134 reflections
147 parameters
H-atom parameters constrained
Δρ_max_ = 0.22 e Å^−3^
Δρ_min_ = −0.18 e Å^−3^

### 

Data collection: *CrysAlis PRO* (Agilent, 2012 ▶); cell refinement: *CrysAlis PRO*; data reduction: *CrysAlis RED* (Agilent, 2012 ▶); program(s) used to solve structure: *SUPERFLIP* (Palatinus & Chapuis, 2007 ▶); program(s) used to refine structure: *SHELXL2012* (Sheldrick, 2008 ▶); molecular graphics: *OLEX2* (Dolomanov *et al.*, 2009 ▶); software used to prepare material for publication: *OLEX2*.

## Supplementary Material

Crystal structure: contains datablock(s) I. DOI: 10.1107/S1600536813028031/su2656sup1.cif

Structure factors: contains datablock(s) I. DOI: 10.1107/S1600536813028031/su2656Isup2.hkl

Supplementary material file. DOI: 10.1107/S1600536813028031/su2656Isup3.cml

Additional supplementary materials:  crystallographic information; 3D view; checkCIF report

## Figures and Tables

**Table 1:** Hydrogen-bond geometry (Å, °)

*D*—H⋯*A*	*D*—H	H⋯*A*	*D*⋯*A*	*D*—H⋯*A*
O2—H2⋯O1^i^	0.82	1.88	2.6953 (14)	170

Symmetry code: (i) 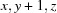.

**Fig. 1.**
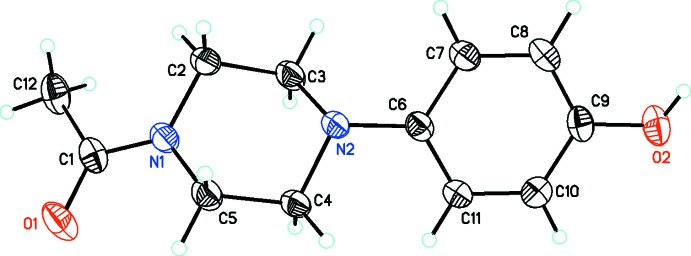
The molecular structure of the title molecule, with atom labelling. Displacement ellipsoids are drawn at the 50% probability level.

**Fig. 2.**
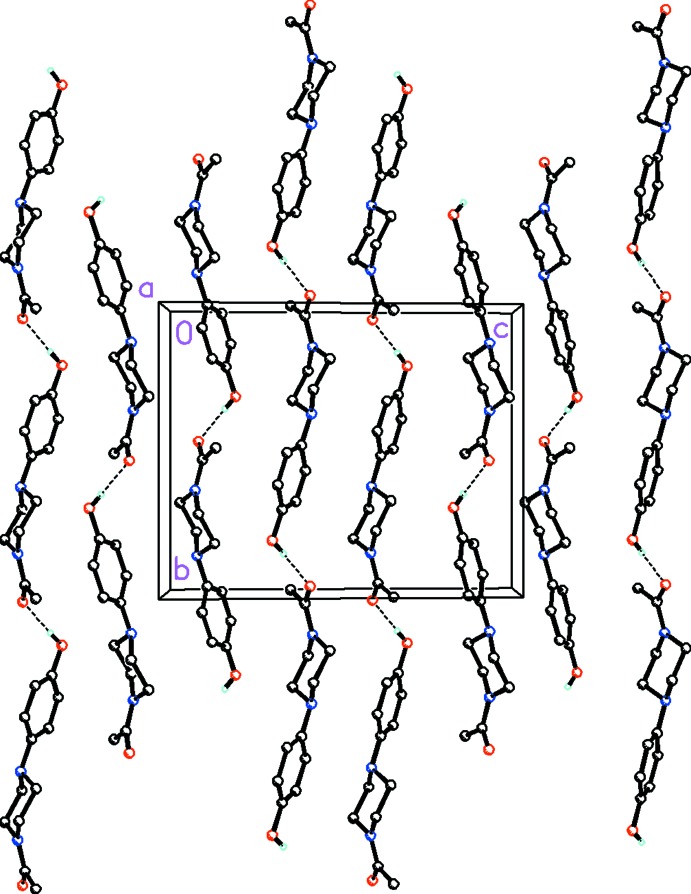
A view along the a axis of the crystal packing of the title compound. The O—H···O hydrogen bonds, linking the molecules into chains along [0 1 0], are shown as dashed lines (see Table 1 for details; H atoms not involved in hydrogen bonding have been omitted for clarity).

## supplementary crystallographic information

### 1. Comment

The title compound is used to synthesize ketoconazole which is a antifungal agent. A valuable insight into recent advances on antimicrobial activity of piperazine derivatives has been reported by (Kharb *et al.*, 2012). Many currently notable drugs contain a piperazine ring as part of their molecular structure. Piperazines are also among the most important building blocks in today's drug discovery and are found in biologically active compounds across a number of different therapeutic areas (Brockunier *et al.*, 2004; Bogatcheva *et al.*, 2006). A review on the current pharmacological and toxicological information for piperazine derivatives is described (Elliott, 2011). The crystal structures of some related compounds, viz., cis-1-acetyl-4-(4-{[2-(2,4-dichlorophenyl)-2-(1H-1-imidazolyl methyl)-1,3-dioxolan-4-yl]methoxy}phenyl) piperazine: ketoconazole. A crystal structure with disorder (Peeters *et al.*, 1979), (+)-cis-1-acetyl-4-(4-{[(2R,4S)-2-(2,4-dichlorophenyl)-2-(1H- imidazol-1-ylmethyl)-1,3-dioxolan-4-yl]methoxy}phenyl)piperazine [(2R,4S)-(+)-ketoconazole] (Peeters *et al.*, 2004), 1-{4-[bis (4-fluorophenyl)methyl]piperazin-1-yl}ethanone (Dayananda *et al.* , 2012), cinnarizinium bis(p-toluenesulfonate)dihydrate (Kavitha *et al.*, 2013*a*) and flunarizinium hydrogen maleate (Kavitha *et al.*, 2013*b*) have been reported. In view of the importance of the title compound this paper reports its crystal structure.

The molecular structure of the title compound is illustrated in Fig. 1. The piperazine ring has a chair conformation with puckering parameters (Cremer & Pople, 1975), Q, θ, and φ = 0.5661 (13) Å, 174.05 (12)° and 0.9 (13)°, respectively. The dihedral angle between the mean planes of the benzene ring (C6-C11) and the acetyl group (N1/C1/C12/O1) is 48.7 (1)°. Bond lengths are in normal ranges (Allen *et al.*, 1987).

In the crystal, O—H···O hydrogen bonds (Table 1) are observed which link the molecules into chains along [0 1 0], as shown in Fig. 2.

### 2. Experimental

The title compound was purchased from Sigma-Aldrich and was recrystallized from ethanol by slow evaporation to give irregular block-like colourless crystals (M.p. = 453 K).

### 3. Refinement

All of the H atoms were placed in calculated positions and refined as riding atoms: C—H = 0.93 Å (CH), 0.97 Å (CH_2_), 0.96 Å (CH_3_), and O-H = 0.82 Å, with U_iso_(H) = 1.5U_eq_(C-methyl and O) and = 1.2U_eq_(C) for other H atoms.

### Crystal data

**Table:**

C_12_H_16_N_2_O_2_	*F*(000) = 472
*M**_r_* = 220.27	*D*_x_ = 1.339 Mg m^−^^3^
Monoclinic, *P*2_1_/*c*	Cu *K*α radiation, λ = 1.54184 Å
*a* = 6.13183 (19) Å	Cell parameters from 3201 reflections
*b* = 12.0106 (4) Å	θ = 3.7–72.1°
*c* = 14.8704 (5) Å	µ = 0.75 mm^−^^1^
β = 94.025 (3)°	*T* = 173 K
*V* = 1092.46 (6) Å^3^	Block, colourless
*Z* = 4	0.48 × 0.46 × 0.32 mm

### Data collection

**Table:**

Agilent Xcalibur (Eos, Gemini) diffractometer	2134 independent reflections
Radiation source: Enhance (Cu) X-ray Source	1944 reflections with *I* > 2σ(*I*)
Detector resolution: 16.0416 pixels mm^-1^	*R*_int_ = 0.025
ω scans	θ_max_ = 72.3°, θ_min_ = 4.7°
Absorption correction: multi-scan (*CrysAlis PRO* and *CrysAlis RED*; Agilent, 2012)	*h* = −7→5
*T*_min_ = 0.833, *T*_max_ = 1.000	*k* = −14→14
6224 measured reflections	*l* = −17→18

### Refinement

**Table:**

Refinement on *F*^2^	0 restraints
Least-squares matrix: full	Hydrogen site location: inferred from neighbouring sites
*R*[*F*^2^ > 2σ(*F*^2^)] = 0.041	H-atom parameters constrained
*wR*(*F*^2^) = 0.113	*w* = 1/[σ^2^(*F*_o_^2^) + (0.063*P*)^2^ + 0.2496*P*] where *P* = (*F*_o_^2^ + 2*F*_c_^2^)/3
*S* = 1.07	(Δ/σ)_max_ < 0.001
2134 reflections	Δρ_max_ = 0.22 e Å^−^^3^
147 parameters	Δρ_min_ = −0.18 e Å^−^^3^

### Special details

**Table:**

Geometry. All esds (except the esd in the dihedral angle between two l.s. planes) are estimated using the full covariance matrix. The cell esds are taken into account individually in the estimation of esds in distances, angles and torsion angles; correlations between esds in cell parameters are only used when they are defined by crystal symmetry. An approximate (isotropic) treatment of cell esds is used for estimating esds involving l.s. planes.

### Fractional atomic coordinates and isotropic or equivalent isotropic displacement parameters (Å^2^)

**Table:**

	*x*	*y*	*z*	*U*_iso_*/*U*_eq_	
O1	0.76473 (18)	−0.03376 (8)	0.41180 (7)	0.0420 (3)	
O2	0.92639 (18)	0.80536 (7)	0.30904 (7)	0.0375 (3)	
H2	0.8626	0.8514	0.3383	0.056*	
N1	0.62601 (17)	0.13923 (9)	0.41758 (7)	0.0273 (3)	
N2	0.71764 (16)	0.37192 (8)	0.41491 (7)	0.0244 (3)	
C1	0.6095 (2)	0.03108 (10)	0.39631 (8)	0.0285 (3)	
C2	0.44858 (19)	0.22001 (10)	0.40349 (9)	0.0299 (3)	
H2A	0.3914	0.2384	0.4608	0.036*	
H2B	0.3309	0.1879	0.3649	0.036*	
C3	0.5312 (2)	0.32459 (10)	0.36038 (9)	0.0305 (3)	
H3A	0.5754	0.3072	0.3006	0.037*	
H3B	0.4143	0.3791	0.3542	0.037*	
C4	0.89577 (19)	0.29095 (10)	0.42176 (8)	0.0262 (3)	
H4A	1.0203	0.3222	0.4569	0.031*	
H4B	0.9407	0.2738	0.3620	0.031*	
C5	0.8215 (2)	0.18508 (10)	0.46652 (9)	0.0290 (3)	
H5A	0.9381	0.1304	0.4680	0.035*	
H5B	0.7897	0.2012	0.5282	0.035*	
C6	0.77423 (19)	0.48149 (10)	0.38735 (8)	0.0238 (3)	
C7	0.6271 (2)	0.56832 (10)	0.39795 (8)	0.0276 (3)	
H7	0.4959	0.5540	0.4235	0.033*	
C8	0.6738 (2)	0.67575 (10)	0.37087 (8)	0.0293 (3)	
H8	0.5713	0.7319	0.3766	0.035*	
C9	0.8717 (2)	0.70046 (10)	0.33537 (8)	0.0275 (3)	
C10	1.0194 (2)	0.61483 (11)	0.32474 (9)	0.0315 (3)	
H10	1.1523	0.6299	0.3008	0.038*	
C11	0.9702 (2)	0.50671 (10)	0.34968 (9)	0.0290 (3)	
H11	1.0700	0.4500	0.3411	0.035*	
C12	0.3937 (2)	−0.01011 (11)	0.35382 (10)	0.0366 (3)	
H12A	0.4010	−0.0892	0.3450	0.055*	
H12B	0.3631	0.0260	0.2967	0.055*	
H12C	0.2796	0.0067	0.3927	0.055*	

### Atomic displacement parameters (Å^2^)

**Table:**

	*U*^11^	*U*^22^	*U*^33^	*U*^12^	*U*^13^	*U*^23^
O1	0.0495 (6)	0.0251 (5)	0.0499 (6)	0.0119 (4)	−0.0068 (5)	−0.0033 (4)
O2	0.0524 (6)	0.0219 (5)	0.0390 (5)	−0.0023 (4)	0.0087 (4)	0.0006 (4)
N1	0.0268 (5)	0.0210 (5)	0.0336 (6)	0.0034 (4)	−0.0010 (4)	−0.0004 (4)
N2	0.0215 (5)	0.0200 (5)	0.0314 (5)	0.0030 (4)	−0.0008 (4)	0.0000 (4)
C1	0.0396 (7)	0.0221 (6)	0.0238 (6)	0.0032 (5)	0.0016 (5)	0.0015 (4)
C2	0.0229 (6)	0.0235 (6)	0.0431 (7)	0.0027 (5)	−0.0002 (5)	−0.0042 (5)
C3	0.0257 (6)	0.0229 (6)	0.0415 (7)	0.0045 (5)	−0.0071 (5)	0.0006 (5)
C4	0.0226 (6)	0.0232 (6)	0.0323 (6)	0.0048 (5)	−0.0021 (5)	0.0008 (5)
C5	0.0281 (6)	0.0233 (6)	0.0348 (6)	0.0034 (5)	−0.0045 (5)	0.0023 (5)
C6	0.0259 (6)	0.0209 (6)	0.0239 (6)	0.0026 (4)	−0.0028 (4)	−0.0013 (4)
C7	0.0286 (6)	0.0249 (6)	0.0294 (6)	0.0048 (5)	0.0037 (5)	−0.0004 (5)
C8	0.0363 (7)	0.0226 (6)	0.0290 (6)	0.0077 (5)	0.0023 (5)	−0.0015 (5)
C9	0.0383 (7)	0.0204 (6)	0.0233 (6)	−0.0009 (5)	−0.0023 (5)	−0.0011 (4)
C10	0.0274 (6)	0.0299 (7)	0.0371 (7)	−0.0019 (5)	0.0024 (5)	0.0008 (5)
C11	0.0246 (6)	0.0235 (6)	0.0386 (7)	0.0041 (5)	0.0013 (5)	−0.0006 (5)
C12	0.0500 (8)	0.0229 (6)	0.0354 (7)	−0.0018 (6)	−0.0080 (6)	−0.0012 (5)

### Geometric parameters (Å, º)

**Table:**

O1—C1	1.2387 (16)	C4—C5	1.5200 (17)
O2—H2	0.8200	C5—H5A	0.9700
O2—C9	1.3681 (15)	C5—H5B	0.9700
N1—C1	1.3391 (16)	C6—C7	1.3950 (17)
N1—C2	1.4618 (15)	C6—C11	1.3943 (18)
N1—C5	1.4651 (16)	C7—H7	0.9300
N2—C3	1.4688 (15)	C7—C8	1.3875 (18)
N2—C4	1.4607 (14)	C8—H8	0.9300
N2—C6	1.4284 (15)	C8—C9	1.3888 (19)
C1—C12	1.5096 (18)	C9—C10	1.3869 (18)
C2—H2A	0.9700	C10—H10	0.9300
C2—H2B	0.9700	C10—C11	1.3896 (18)
C2—C3	1.5136 (18)	C11—H11	0.9300
C3—H3A	0.9700	C12—H12A	0.9600
C3—H3B	0.9700	C12—H12B	0.9600
C4—H4A	0.9700	C12—H12C	0.9600
C4—H4B	0.9700		
			
C9—O2—H2	109.5	N1—C5—H5A	109.5
C1—N1—C2	124.54 (11)	N1—C5—H5B	109.5
C1—N1—C5	121.83 (10)	C4—C5—H5A	109.5
C2—N1—C5	113.35 (10)	C4—C5—H5B	109.5
C4—N2—C3	109.27 (9)	H5A—C5—H5B	108.0
C6—N2—C3	113.18 (9)	C7—C6—N2	118.97 (11)
C6—N2—C4	115.97 (9)	C11—C6—N2	123.30 (10)
O1—C1—N1	121.45 (13)	C11—C6—C7	117.74 (11)
O1—C1—C12	120.76 (12)	C6—C7—H7	119.5
N1—C1—C12	117.78 (11)	C8—C7—C6	120.95 (12)
N1—C2—H2A	109.6	C8—C7—H7	119.5
N1—C2—H2B	109.6	C7—C8—H8	119.6
N1—C2—C3	110.09 (10)	C7—C8—C9	120.83 (11)
H2A—C2—H2B	108.2	C9—C8—H8	119.6
C3—C2—H2A	109.6	O2—C9—C8	122.96 (11)
C3—C2—H2B	109.6	O2—C9—C10	118.35 (12)
N2—C3—C2	110.98 (10)	C10—C9—C8	118.68 (11)
N2—C3—H3A	109.4	C9—C10—H10	119.8
N2—C3—H3B	109.4	C9—C10—C11	120.45 (12)
C2—C3—H3A	109.4	C11—C10—H10	119.8
C2—C3—H3B	109.4	C6—C11—H11	119.3
H3A—C3—H3B	108.0	C10—C11—C6	121.31 (11)
N2—C4—H4A	109.7	C10—C11—H11	119.3
N2—C4—H4B	109.7	C1—C12—H12A	109.5
N2—C4—C5	109.99 (10)	C1—C12—H12B	109.5
H4A—C4—H4B	108.2	C1—C12—H12C	109.5
C5—C4—H4A	109.7	H12A—C12—H12B	109.5
C5—C4—H4B	109.7	H12A—C12—H12C	109.5
N1—C5—C4	110.90 (10)	H12B—C12—H12C	109.5
			
O2—C9—C10—C11	−179.37 (11)	C4—N2—C6—C7	−166.26 (11)
N1—C2—C3—N2	−56.20 (14)	C4—N2—C6—C11	14.00 (16)
N2—C4—C5—N1	56.23 (13)	C5—N1—C1—O1	−4.88 (19)
N2—C6—C7—C8	−179.02 (11)	C5—N1—C1—C12	174.25 (11)
N2—C6—C11—C10	−179.29 (11)	C5—N1—C2—C3	52.56 (14)
C1—N1—C2—C3	−133.46 (13)	C6—N2—C3—C2	−168.28 (10)
C1—N1—C5—C4	132.86 (12)	C6—N2—C4—C5	170.42 (10)
C2—N1—C1—O1	−178.37 (12)	C6—C7—C8—C9	−2.28 (19)
C2—N1—C1—C12	0.76 (18)	C7—C6—C11—C10	0.97 (19)
C2—N1—C5—C4	−52.98 (14)	C7—C8—C9—O2	−178.98 (11)
C3—N2—C4—C5	−60.23 (13)	C7—C8—C9—C10	2.06 (19)
C3—N2—C6—C7	66.31 (14)	C8—C9—C10—C11	−0.36 (19)
C3—N2—C6—C11	−113.43 (13)	C9—C10—C11—C6	−1.2 (2)
C4—N2—C3—C2	60.86 (13)	C11—C6—C7—C8	0.74 (18)

### Hydrogen-bond geometry (Å, º)

**Table:**

*D*—H···*A*	*D*—H	H···*A*	*D*···*A*	*D*—H···*A*
O2—H2···O1^i^	0.82	1.88	2.6953 (14)	170

Symmetry code: (i) *x*, *y*+1, *z*.
